# Gamma Oscillations and Coherence Are Weaker in the Dorsomedial Subregion of STN in Parkinson's Disease

**DOI:** 10.3389/fneur.2021.710206

**Published:** 2021-09-07

**Authors:** Jing Wei, Zhifan Zou, Jiping Li, Yuqing Zhang

**Affiliations:** ^1^School of Biomedical Engineering, Capital Medical University, Beijing, China; ^2^Department of Physiology and Pathophysiology, Capital Medical University, Beijing, China; ^3^Beijing Institute of Functional Neurosurgery, Xuanwu Hospital, Capital Medical University, Beijing, China

**Keywords:** Parkinson's disease, deep brain stimulation, subthalamic nucleus, local field potentials, gamma activity

## Abstract

**Background:** Deep-brain stimulation (DBS) of the subthalamic nucleus (STN) is an effective treatment for motor symptoms of advanced Parkinson's disease (PD). Due to a lack of detailed somatotopic organization in STN, the clinically most effective part of the STN for stimulation has already become one of the hot research focuses. At present, there are some reports about topographic distribution for different depths within the STN, but few about a mediolateral topography in this area.

**Objective:** The objective was to investigate the local field potential (LFP) distribution patterns in dorsomedial and dorsolateral subparts of STN.

**Methods:** In total, 18 PD patients eventually enrolled in this study. The DBS electrodes were initially located on the lateral portion of dorsolateral STN. Because of internal capsule side effects presented at low threshold (below 1.5 mA), the electrode was reimplanted more medially to the dorsomedial STN. In this process, intraoperative LFPs from dorsomedial and dorsolateral STN were recorded from the inserted electrode. Both beta power and gamma power of the LFPs were calculated using the power spectral density (PSD) for each DBS contact pair. Furthermore, coherence between any two pairs of contacts was computed in the dorsomedial and dorsolateral parts of STN, respectively. Meanwhile, the Unified Parkinson's Disease Rating Scale part III (UPDRS-III) was monitored prior to surgery and at the 6-month follow-up.

**Results:** Compared to the dorsolateral part of STN, gamma oscillations (*p* < 0.01) and coherence (*p* < 0.05) were all weaker in the dorsomedial part. However, no obvious differences in beta oscillations and coherence were observed between the two groups (*p* > 0.05). Moreover, it should be noted that DBS of the dorsomedial STN resulted in significant improvement in the UPDRS-III in PD patients. There was a 61.50 ± 21.30% improvement in UPDRS-III scores in Med-off/Stim-on state relative to the Med-off state at baseline (from 15.44 ± 6.84 to 43.94 ± 15.79, *p* < 0.01).

**Conclusions:** The specific features of gamma activity may be used to differentiate STN subregions. Moreover, the dorsomedial part of STN might be a potential target for DBS in PD.

## Introduction

Parkinson's disease (PD) is a movement disorder resulting from the degeneration of dopaminergic neurons in the pars compacta of the substantia nigra (SNc), which is characterized by pathological oscillatory activity in the cortico-basal ganglia circuit.

Synchronized oscillations have been hypothesized to be one key mechanism for communication among different neuronal populations (1). Researchers have proven that prominent beta band oscillations from the subthalamic nucleus (STN) were observed in PD patients (2), which was presumed to be correlated with the motor impairment in PD (3, 4). Then, it has been reported that beta activity was attenuated by levodopa and during high-frequency deep-brain stimulation (DBS), accompanied by clinical symptom improvement (5, 6). Therefore, beta activity has been supposed to inhibit movement and play a key role to bradykinesia and rigidity in PD (4, 7). In contrast, gamma activity has been labeled “prokinetic” which is increased during movement (8). Pronounced increases in gamma oscillations, especially the finely tuned gamma (FTG), are often observed in PD patients with anti-parkinsonian medication or therapeutic DBS (9–11). Based on these findings, it can be inferred that the therapeutic effects were achieved through rebalancing the hypokinetic and hyperkinetic rhythm in abnormal neural circuits (12, 13).

STN-DBS is a powerful treatment for advanced PD, which has been shown to improve the primary motor symptoms of PD, such as bradykinesia, tremor, and rigidity (14). Nevertheless, the occurrence of side effects may impede the benefit of the clinical outcome. Due to a lack of detailed somatotopic organization in STN, the most clinically effective part of the STN for stimulation has already become one of the hot research focuses. At present, there are some reports about topographic distribution for different depths within the STN, but few about a mediolateral topography in the STN. Studies have suggested that the dorsolateral motor part of the STN may be an optimal target for DBS where it produces maximum clinical effects and minimum side effects (15, 16). Telkes et al. presented an automated method to estimate the optimal track for the DBS electrode by combining different frequency characteristics for different depths within STN (17). Another study found that depth-specific temporal spike patterns of single neurons in STN had connections with the optimal DBS target location for tic suppression in Tourette syndrome (18).

In our study, the DBS electrode located in the dorsolateral STN of some PD patients was so close to the internal capsule that is currently spread into this unwanted anatomical area, resulting in eye deviation, tonic limb, or face contraction. In this case, the DBS electrode should be considered to reposition and retest to reduce the side effects. For this work, the electrode was medially repositioned by 2–3 mm and finally placed in the dorsomedial region of STN. Thus, there is an opportunity to obtain the pathological neural activity in both dorsomedial and dorsolateral STN *via* intraoperative local field potential (LFP) recordings. What is more, the effect of dorsomedial STN DBS for PD could be investigated.

## Materials and Methods

### Patients

All subjects were recruited from the functional neurosurgery department of the Xuanwu Hospital of Capital Medical University. Inclusion criteria were (a) age 18–75 years; (b) met the MDS clinical diagnostic criteria of PD (2), H-Y stages 2–4, with motor fluctuation and/or dyskinesia; (c) MMSE score more than 24; (d) no history of cerebrovascular disease, seizures, and psychiatric disorders; and (e) internal capsule side effects presented at the low threshold (below 1.5 mA) during the intraoperative macrostimulation, as well as intraoperative CT/MRI infusion confirming that the electrode was located on the lateral portion of dorsolateral STN. Then the electrode was reimplanted more medially to the dorsomedial STN, which is confirmed by postoperative CT/MRI infusion. This experiment was approved by the institutional review board of Xuanwu Hospital. All the participants were provided written informed consent prior to the experiment.

### STN-DBS Surgical Procedure

STN-DBS electrode implantation was performed under local anesthesia after overnight withdrawal of medication. A CT scan with head frame (CRW stereotactic frame, Radionics, Webster, NY, USA) and fusion with the preoperative magnetic resonance imaging (MRI; Siemens 3.0, Tesla, Sonata, Germany) images through the Stealth Station surgical navigation system (Medtronic, Minneapolis, MN, USA) were performed on the operating day, with the STN target based on the midcommissural point at the following coordinates: (X) 12 mm lateral, (Y) 2 mm posterior, and (Z) 4 mm inferior, targeted at its dorsolateral area. Intraoperative microelectrode single-needle recording (MER) using the Microdrive system (Alpha Omega Engineering, Nazareth, Israel) was performed, starting from 10 mm above to 5 mm below the STN target to confirm the ventral margin of STN. DBS electrodes (Model 3389, Medtronic, Minneapolis, MN, USA) were placed as a metal tip located at the ventral margin, then bipolar LFPs were recorded using the EEG monitoring system (Micromed, Treviso, Italy) from the inserted DBS leads for about 60 s. During the LFP recordings, patients were awake and instructed to keep their eyes opened and rest without voluntary movement or speech. Then, macrostimulation was used to observe the DBS efficacy and side effects after withdrawing the tip of the DBS lead back to the target point. The stimulation parameters were as follows: unipolar stimulation (C+0−), frequency 130 Hz, and pulse width 90 μs. The amplitudes were from 0.5 to 5 mA or when patients present side effects. If internal capsule side effects presented at low threshold (below 1.5 mA), CT scanning and fusion with the preoperative MRI images were performed immediately to confirm the electrode position. If the electrode was located on the lateral portion of dorsolateral STN, then the inserted DBS electrode was pulled out and reimplanted 2 mm more medial, the MER was repeated, and LFP recording and macrostimulation were performed. After a satisfactory outcome of macrostimulation was obtained, the DBS electrodes were internalized and connected to an impulse generator implanted in the subclavian region under general anesthesia. Postoperative CT images were fused with the preoperative MRI images to confirm the position of electrodes.

### Data Acquisition and Preprocessing

LFPs were recorded bipolarly from adjacent contacts (0–1, 1–2, 2–3) of the inserted DBS lead. All recordings were sampled at 256 Hz. First, the raw data were high-pass filtered at 1 Hz to avoid effects of low-frequency direct current fluctuations. Then, power line noise (50 Hz) and its harmonics were notch filtered from the data using a Butterworth filter. Finally, the resulting data were used for further LFP analysis, which was performed offline in Matlab (version 9.1).

### Time–Frequency Analysis

To investigate the LFP dynamics in the time–frequency domain, the time–frequency spectrum was calculated by the short-time Fourier transform (STFT) with the multi-taper analysis. The sliding time window was 1 s and the time step was 0.25 s, yielding a frequency resolution of 1 Hz. The absolute power spectra, which were estimated by the power spectral density (PSD), were then transformed to a logarithmic scale and shown in decibels (dB). The above time–frequency analysis was applied to the LFPs of all contact pairs from each trial.

### Power Spectrum Analysis

The power spectra were averaged over the frequencies of interest. In this work, two frequency bands were considered: beta band (13–30 Hz) and gamma band (31–48, 52–98, 102–122 Hz). As the study focuses on LFP activity in different subregions of the STN, the spectral value was averaged across three contact pairs (0–1, 1–2, and 2–3) of a DBS electrode.

### Coherence Analysis

To evaluate the LFP interactions in STN, coherence was calculated between any two LFPs from neighboring electrode contact pairs. Coherence between signal *x* and signal *y* was computed according to the following formula:

$$\begin{array}{l} C_{xy}\left(f\right)=\frac{|P_{xy}\left(f\right){|}^{2}}{P_{xx}\left(f\right)P_{yy}\left(f\right)} \end{array}$$

Here, *P*_*xy*_ is the cross power spectral density between *x* and *y*, and *P*_*xx*_ and *P*_*yy*_ are the individual power spectral densities of x and y, respectively. The magnitude coherence *C*_*xy*_ is a function of frequency, whose values are between 0 and 1. For a given frequency, 0 indicates that the two signals are independent and 1 indicates their amplitudes co-vary.

For beta and gamma bands, the coherence results were obtained by averaging the coherence values over their own frequency bands. Moreover, for a given DBS electrode, the final coherence value was averaged across all three contact pairs (coherence between 0–1 and 1–2, between 1–2 and 2–3, between 0–1 and 2–3).

### Statistical Analysis

Statistical analyses were performed by using SPSS 21.0 software. All data were expressed as mean ± standard deviation (SEM), unless otherwise specified.

The assessments of Unified Parkinson's Disease Rating Scale part III (UPDRS III) were completed in both Med-off and Med-on states at baseline and in DBS stimulation-on (Stim-on) condition at 6 months of follow-up after surgery. The clinical improvement was computed as {[(Prescores – Postscores) / Prescores] ^*^100%}. Appropriate statistical tests were used to determine whether there was a significant difference between the UPDRS III scores at baseline and at 6 months of follow-up. In addition, the corresponding statistical analysis has been computed for LFP oscillation and coherence results.

The specific method was as follows: First, the Kolmogorov–Smirnov test was applied to examine the normality of measurement data distribution. Then, for the normally distributed data, two-tailed independent-sample *t*-tests were used. Instead, if normality could not be assumed in data, nonparametric testing (Mann–Whitney U) was used. The level of significance was set to *p* < 0.05 for all statistical analyses.

## Results

In total, 303 PD patients underwent STN DBS surgery between January 2017 and December 2018. Eventually, 18 patients (5.94%) were included in this study. Although all these 18 patients were bilateral STN-DBS implantation, the electrode reimplantation took place only on one side of the brain, so the LFP was also recorded in this side. Of the 18 patients, 7 were female and 11 were male. Their mean age was 55.30 ± 9.11 years. The mean duration of disease before the surgery was 9.83 ± 4.15 years. The preoperative levodopa equivalent daily dose (LEDD) was 749.78 ± 248.10 mg/day. There was a 57.30 ± 14.45% improvement in UPDRS-III scores in the Med-on state relative to the Med-off state at baseline. At the 6-month follow-up, there was a 61.50 ± 21.30% improvement in UPDRS-III scores in the Med-off/Stim-on state relative to the Med-off state at baseline (from 15.44 ± 6.84 to 43.94 ± 15.79, *p* < 0.01) and a 76.17 ± 16.48% improvement in the Med-on/Stim-on state relative to the Med-off state at baseline (from 9.40 ± 5.41 to 43.94 ± 15.79, *p* < 0.01). The demographics are presented in Table 1. It needs to be reminded that all behavioral results were expressed as mean ± standard deviation (SD).

**Table 1:** Patient demographic and clinical characterization.

**Patient number**	**Age (years)**	**Disease duration (years)**	**Daily L-DOPA equivalent dose (mg)**	**Preoperative** **UPDRS part III**		**Postoperative** **UPDRS part III**	
				**OFF**	**ON**	**OFF**	**ON**
PD 1	45	16	801	74	20	25	5
PD 2	34	6	700	28	8	9	7
PD 3	62	10	723	40	14	13	9
PD 4	60	13	1220	30	8	18	15
PD 5	71	14	750	48	20	13	10
PD 6	57	20	963	71	22	21	10
PD 7	47	8	300	28	15	4	3
PD 8	64	10	800	54	31	9	10
PD 9	45	7	226	73	29	10	6
PD 10	56	6	375	43	12	14	6
PD 11	54	4	938	31	19	13	2
PD 12	45	8	1000	40	14	11	7
PD 13	60	11	675	47	33	7	5
PD 14	61	12	900	27	17	15	7
PD 15	66	10	725	35	21	21	10
PD 16	57	5	900	26	11	25	17
PD 17	55	6	700	50	20	23	18
PD 18	57	11	800	46	13	27	22

### STN Oscillatory Activity in the Dorsomedial and Dorsolateral Parts

**Figure 1:**
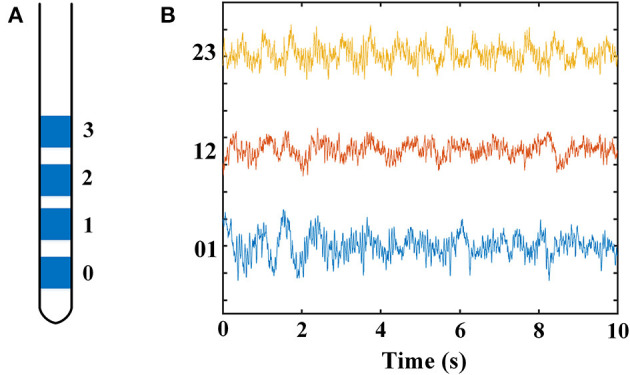
Schematic diagram of a DBS electrode and LFP recordings from a DSB electrode in STN. **(A)** Schematics showing the quadripolar electrode from the anterior view. Contacts 0 and 3 indicating the contacts at bottom and top, respectively. **(B)** Example of LFP signals from all three contact pairs of an electrode located in STN. The X-axis represents time (s), and the Y-axis represents the names of the three contact pairs. Blue line: LFP from contact pair 0–1; red line: LFP from contact pair 1–2; yellow line: LFP from contact pair 2–3. DBS, deep brain stimulation; LFP, local field potential; STN, subthalamic nucleus.

The contacts of a DBS electrode were numbered 0, 1, 2, and 3, with 0 being the most ventral and 3 the most dorsal contact (Figure 1A). Figure 1B demonstrates an example of LFPs recorded from adjacent contacts of an electrode. The mean durations of recordings were 76.2 ± 26.4 s and 67.1 ± 14.3 s (mean ± SD) for the dorsomedial and dorsolateral parts of STN, respectively. There was no significant difference in the recording length for LFPs for the above two groups (*p* > 0.05).

**Figure 2:**
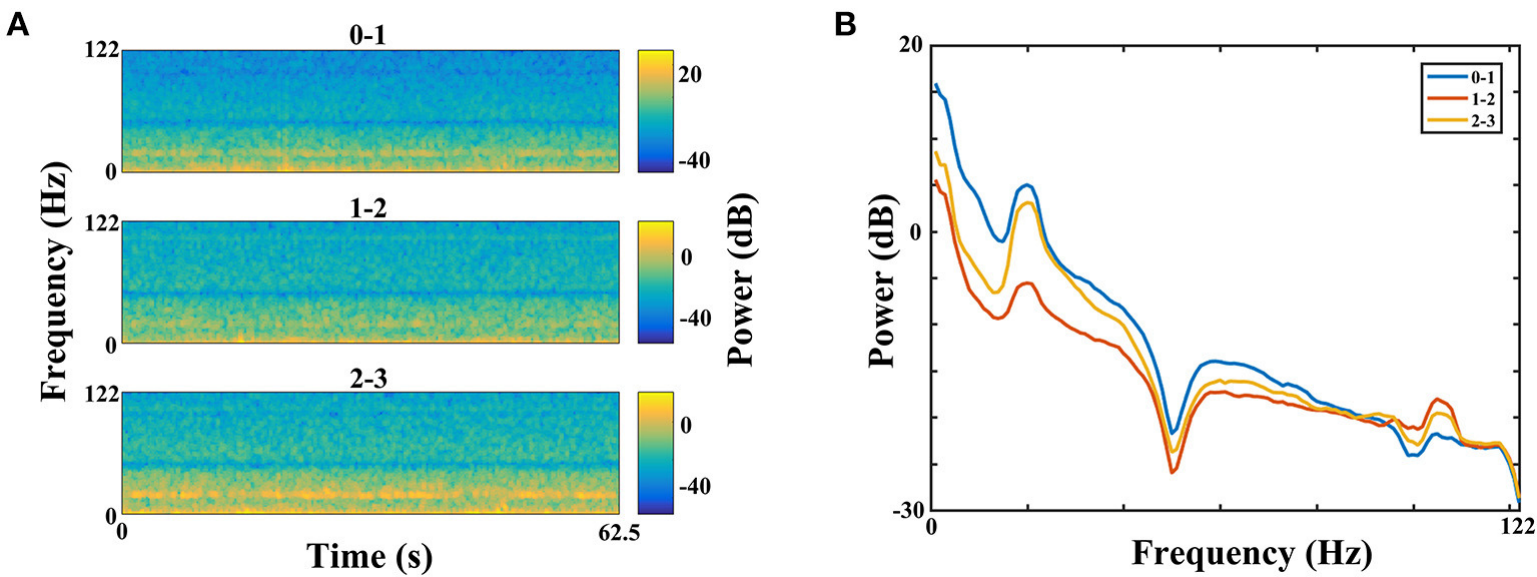
Time–frequency spectrograms **(A)** and PSDs **(B)** of LFPs from all three electrode pairs (0–1, 1–2, and 2–3) of STN in a representative patient (B, blue = 0–1, red = 1–2, yellow = 2–3). **(A)** The spectrogram has one axis (X-axis) for the time domain (s) and one axis (Y-axis) for the frequency domain (Hz) and uses color to represent power levels (dB). The color legend is displayed to the right of each spectrogram. Visual inspection of the maps indicated distinct beta band (13–30 Hz) and less obvious gamma band (100–110 Hz) activity. **(B)** The PSDs indicated that increased amplitudes of beta and gamma oscillations were present in this subject. The X-axis represents frequency (Hz), and the Y-axis represents power (dB). Blue line: PSD from contact pair 0–1; red line: PSD from contact pair 1–2; yellow line: PSD from contact pair 2–3. PSD, power spectral density; LFP, local field potential; STN, subthalamic nucleus.

Figure 2 displays the time–frequency spectrogram and PSD of LFP recordings in STN, from a representative patient. Figure 2A illustrates the spectrogram results from all three pairs of an electrode, respectively. Visual inspection of the maps indicated distinct beta band (13–30 Hz) and less obvious gamma band (100–110 Hz) activity. In order to examine the spectral dynamics, the power spectra for each contact pair were computed, which are shown in Figure 2B. Likewise, increased amplitudes of beta and gamma oscillations were present in this subject. However, the increase of gamma power was found in some, but not all, of dorsomedial and dorsolateral STN. The percent in each group with this increased oscillation pattern was 33.3% (dorsomedial region) and 72.2% (dorsolateral region), respectively.

**Figure 3:**
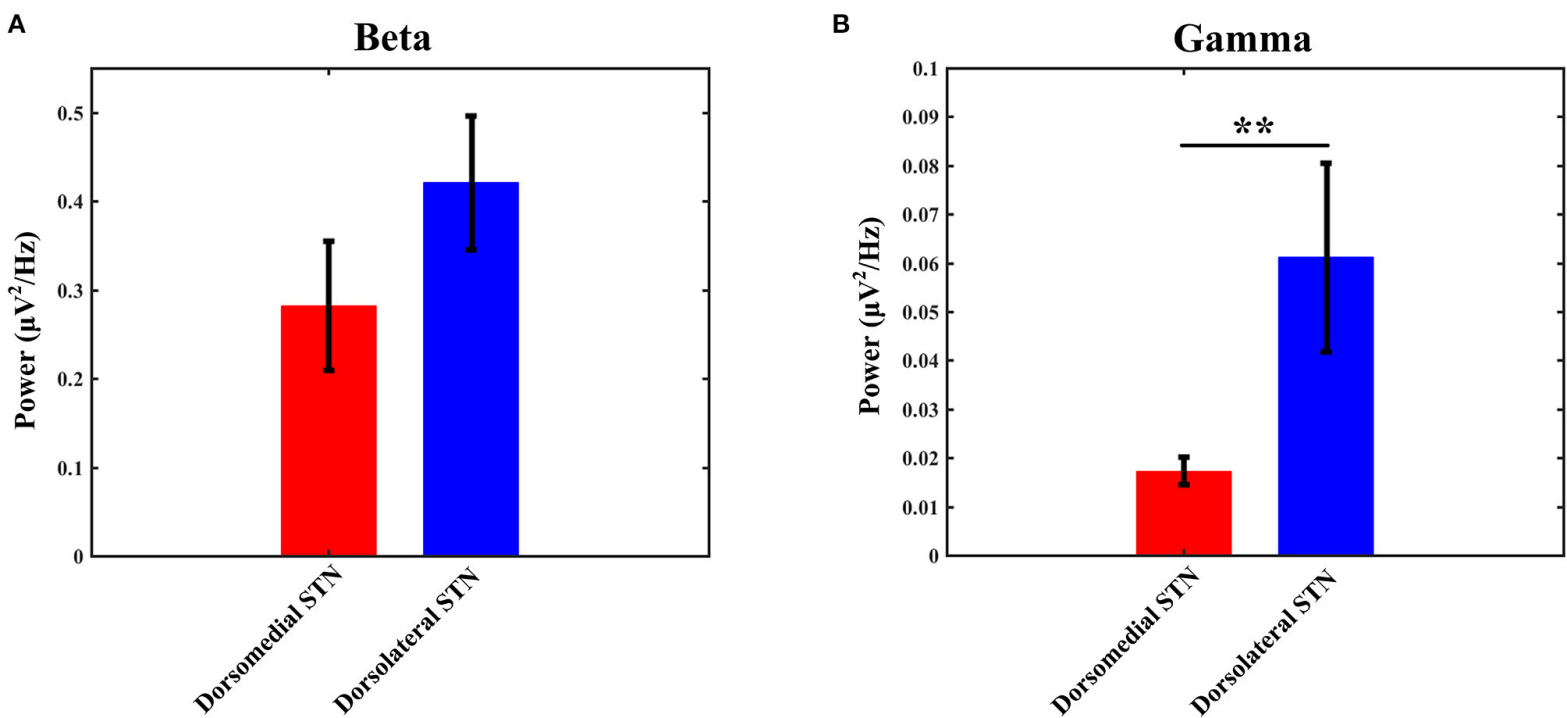
Comparison of beta **(A)** / gamma **(B)** power between the dorsomedial and dorsolateral STNs from 18 patients (36 sides). The power for each trial represents the average of the power values across all valid pairs per side. Red bar = dorsomedial STN, blue bar = dorsolateral STN. Error bars represent standard error. Moreover, **denotes statistical significance (*p* < 0.01). STN: subthalamic nucleus.

The power of each STN subregion was obtained by averaging power across all available electrode pairs. The mean value of beta power in the dorsomedial group was 0.282 ± 0.073, and the corresponding value in the dorsolateral group was 0.421 ± 0.075. The statistical result showed that there was no difference in beta power between the dorsomedial and dorsolateral STN (*p* > 0.05, Figure 3A). However, for gamma activity, the mean value in the dorsomedial group was 0.0173 ± 0.003 and the corresponding value in the dorsolateral group was 0.0611 ± 0.019. It should be noted that the dorsolateral STN exhibited significantly greater spectral power than the dorsomedial STN (*p* < 0.01, Figure 3B).

### STN Coherence in the Dorsomedial and Dorsolateral Parts

**Figure 4:**
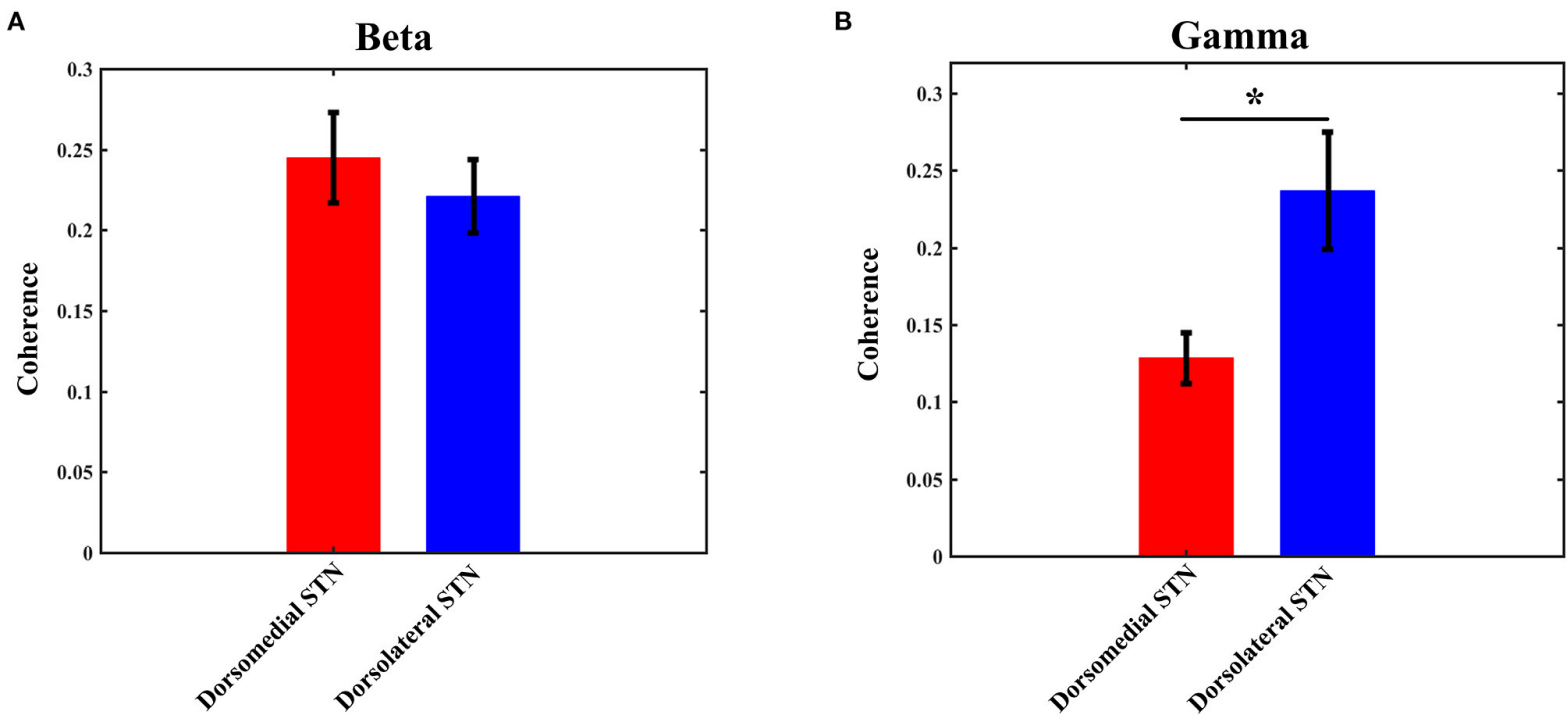
Average beta **(A)** / gamma **(B)** coherence for the dorsomedial (red) and dorsolateral (blue) STNs. *Indicates *p* < 0.05. STN, subthalamic nucleus.

The coherence values of beta/gamma-band LFP between any two pairs of contacts (0–1 and 1–2, 1–2 and 2–3, 0–1 and 2–3) were computed with the method as described in the materials and methods section. Then, the three computed values were averaged to obtain the final coherence for each electrode, which was used to measure the interaction in any subregion of STN. Similar to the power results, statistical analysis did not show any significant difference with beta-band coherence between the dorsomedial and dorsolateral STN (Figure 4A): the mean value of beta coherence in the dorsomedial group was 0.245 ± 0.028, and the corresponding measure in the dorsolateral group was 0.221 ± 0.023 (*p* > 0.05). Notably, there was a significant difference in gamma band coherence, with lower gamma coherence in the dorsomedial STN, compared to the dorsolateral subregion (Figure 4B, *p* < 0.05). The mean value of gamma coherence in the dorsomedial group was 0.128 ± 0.016, and the same measure in the dorsolateral group was 0.237 ± 0.038.

## Discussion

During DBS electrode implantation surgery, LFPs from the dorsomedial and dorsolateral portions of unilateral STN in 18 PD patients were recorded and analyzed in this study. Two major findings in this study are the following: (1) there was no difference in beta oscillation and coherence between the dorsomedial and dorsolateral subregions of STN; (2) however, gamma activity (oscillation and coherence) was lower in the dorsomedial STN, compared with that of the dorsolateral subparts.

### There Was No Difference of the Beta Activity (Both Oscillation and Coherence) Between the Dorsomedial and Dorsolateral STN

Previous research indicates that abnormally enhanced beta oscillations in PD might limit the capacity for neural information coding and processing within the cortico-basal ganglia circuit (19, 20). Some studies have shown that an elevated coherence within the beta band occurred between the pallidum and cortex and also between the STN and cortex, which was presumed to be related to the pathophysiological changes in PD (21–23). Evidence from a study suggests that higher beta synchronization which is measured by phase coherence exists in dorsal STN. Moreover, this pathological synchronization positively correlates with severity of bradykinesia and rigidity in PD patients off medication (24). However, no such relationship is found between beta activity and severity of PD motor impairment (25).

According to intraoperative electrophysiological measurements, neurons with beta frequency band activity and peak beta power were also localized in the dorsolateral motor part of the STN, which was proved as the most appropriate DBS target to achieve favorable clinical results (25–28). However, the internal capsule is adjacent to the lateral part of STN. For some PD patients, if the distance between the position of the DBS electrode in dorsolateral STN and the internal capsule was too close, it might produce side effects during STN DBS programming. The researchers speculated that current spread to pyramidal tract (PT) fibers in internal capsule may disrupt the pattern of information transmission along the corticospinal and corticobulbar pathways (29). In this study, the internal capsule adverse effects happened at low stimulation amplitudes during intraoperative macrostimulation; thus, the DBS electrodes were medially repositioned by 2 mm. In this process, LFPs in both of dorsomedial and dorsolateral STN were recorded and analyzed. The beta activity (oscillation and coherence) from the very lateral portion of dorsolateral STN was compared with that from the dorsomedial STN; however, there was no marked difference between them. In fact, the LFPs have only been recorded through implanted macroelectrodes and so it is not clear whether the results of all regions of dorsolateral STN were in keeping with our findings, which needs to be further studied. Yet, it was worth noting that the ideal clinical effect was obtained in our study although the electrode was located in dorsomedial STN. Therefore, the dorsomedial part of STN might be a potential target for DBS in PD.

### Gamma Activity (Both Oscillation and Coherence) Was Significantly Weaker in the Dorsomedial STN, Compared With That in the Dorsolateral Subregion

As the study went on, some interesting differences in another movement-related activity, gamma frequency activity, were shown. With the development of research, it has been shown that beta and gamma oscillations were a pair of neural activity with antagonistic interaction on each other (13). Further research also indicated that both levodopa and DBS worked by reducing beta band synchronization while promoting gamma band synchronization in the motor network (19, 30, 31). A study suggested that both gamma power and burst rate in STN correlated negatively with parkinsonian motor impairment (32). Still, there was evidence that increased gamma activity in STN was observed when motor performances of PD patients were improved during their ON state (10). Another study suggested that the detected increase in gamma oscillations might make compensation for the pathological increase in beta activity, which inhibited a continuous movement, such as progressive bradykinesia in PD (33). In summary, gamma frequencies may facilitate motor performance during voluntary movements.

Earlier research had found that gamma activity was greater in the upper STN and bordering zona incerta (34). Further study showed that low gamma oscillations and coherence in dorsal STN increased during stronger tremor in PD patients, implicating that this activity may be involved in sensorimotor function. The study also found that the gamma activity could have an effect on downstream neurons in STN; it would be assumed that abnormally enhanced gamma activity may contribute to tremor generation indirectly (35). In our study, gamma oscillations in the OFF medication state were recorded at rest whose amplitudes were smaller than that of beta activity. The results showed that the baseline levels of gamma activity were lower in the dorsomedial STN, compared with that of the dorsolateral subparts. Studies have indicated that the dorsolateral sector of the STN receives afferent projections from the primary motor cortex (M1). Moreover, the supplementary motor area (SMA) projects to the dorsomedial part of the STN. The sensory inputs from the SMA were weaker than those from the M1 (36). Due to the different anatomical projections, the two subregions of STN might contribute differently to movement function. This might be the reason why gamma activity was so different between the dorsomedial and dorsolateral parts of STN.

Studies have suggested that gamma activity increases during voluntary movement and after dopaminergic therapy (13). Further analysis in this study showed that an increase of gamma oscillation was found in some, but not all, of dorsomedial and dorsolateral STN. The percent in each group with this increased oscillation pattern was 33.3% (dorsomedial region) and 72.2% (dorsolateral region), respectively. Based on the previous research, we hypothesized that the presence of enhanced gamma activity in the OFF medication state might be associated with microlesion effect due to DBS (34).

### Limitations

The first limitation of this study was that the LFP activity was invasively recorded in patients with PD, so corresponding LFP data from healthy control could not be obtained to find the difference between the two groups. Another limitation was that the findings from intraoperative LFP recordings may be affected by the effects of micro-lesion. It was found that gamma activity could affect the distribution of neuronal spiking which was modulated to fire at a specific oscillation phase (34, 37). The coordination between neuronal activity and exaggerated gamma oscillations was inferred to play a role to cause choreiform movements (19). However, in this study, the relationship between gamma activity and single-unit discharge failed to be studied because of lack of spike signals, which could not be recorded simultaneously by macroelectrodes. The third limitation was that the postoperative 6-month follow-up is relatively short, so a longer follow-up period and more case studies are needed to determine the benefits and the risks of this procedure for future cases. The last limitation was that due to the lack of postoperative LFPs by DBS macroelectrodes, it was hard to obtain the pattern of LFP changes in response to the DBS.

## Conclusions

This study has shown that gamma oscillations and gamma coherence were lower in the dorsomedial subregion of STN, while there was no difference in the corresponding results of beta activity between the dorsomedial and dorsolateral STN. These results have a possible implication that the specific feature of gamma activity could prove useful in differentiating STN subregions and the dorsomedial part of STN might be a potential target for DBS in PD.

## Data Availability Statement

The original contributions presented in the study are included in the article/supplementary material, further inquiries can be directed to the corresponding author/s.

## Ethics Statement

The studies involving human participants were reviewed and approved by the ethics committee of Xuanwu Hospital of Capital Medical University. The patients/participants provided their written informed consent to participate in this study.

## Author Contributions

JW was the major contributor in writing the manuscript. JL and YZ contributed to DBS surgery and data acquisition. JW and ZZ contributed to data analysis. YZ was the guarantor of integrity of the entire study. All the authors have collectively poured a lot of effort into this study, read, and approved the final manuscript.

## Funding

JW was supported by the National Natural Science Foundation of China (61701323). This study was also supported by a research grant from Medtronic Inc, Minneapolis, MN, USA.

## Conflict of Interest

The authors declare that the research was conducted in the absence of any commercial or financial relationships that could be construed as a potential conflict of interest.

## Publisher's Note

All claims expressed in this article are solely those of the authors and do not necessarily represent those of their affiliated organizations, or those of the publisher, the editors and the reviewers. Any product that may be evaluated in this article, or claim that may be made by its manufacturer, is not guaranteed or endorsed by the publisher.
